# Magic extraction: solid-phase extraction and analytical pyrolysis to study polycyclic aromatic hydrocarbon and polychlorinated biphenyls in freshwater

**DOI:** 10.1007/s11356-022-22435-9

**Published:** 2022-08-08

**Authors:** Jacopo La Nasa, Greta Biale, Francesca Modugno, Alessio Ceccarini, Stefania Giannarelli

**Affiliations:** 1 Department of Chemistry and Industrial Chemistry, University of Pisa, Pisa, Italy; 2 National Interuniversity Consortium of Materials Science and Technology (INSTM), Florence, Italy

**Keywords:** PCBs, PAHs, Pyrolysis, Gas chromatography, Mass spectrometry, Thermal desorption

## Abstract

Polycyclic aromatic hydrocarbons and polychlorinated biphenyls are commonly categorized as persistent organic pollutants. In order to analyze these pollutants, customized stationary phases are increasingly being developed and synthesized for solid-phase extraction. In this work, we tested a new solventless solid-phase extraction approach based on the use of a Magic Chemisorber® (Frontier Lab) which consists of a bead-covered polydimethylsiloxane stationary phase with a thickness of 500 µm. These devices are directly immersed into aqueous samples and then introduced into a pyrolysis–gas chromatography-mass spectrometry system equipped with a cryofocusing system for the thermal desorption and analysis of the adsorbed species. Our new method performs better than the most recent solid-phase extraction devices, with limits of detection lower than 2.7 ng/L and limits of quantification lower than 9.0 ng/L. The method was tested on standard compounds and on an environmental sample, showing the potential to characterize other chemical species besides the persistent organic pollutants, such as phthalate plasticizers and antioxidants.

**Supplementary Information:**

The online version contains supplementary material available at 10.1007/s11356-022-22435-9.

## Introduction

Polycyclic aromatic hydrocarbons (PAHs) and polychlorinated biphenyls (PCBs) are classes of compounds that are commonly categorized as persistent organic pollutants (POPs) (Fitzgerald and Wikoff 2014, Huo et al. 2017; Wu et al. 2021). These compounds are present in most environmental compartments especially water, soil, and air (Huang et al. 2020, Manoli and Samara 1999, Qu et al. 2019, Vane et al. 2014). PAHs and PCBs are hydrophobic, environmentally stable chemicals that tend to bioaccumulate, with toxic effects on animal and human health (Drábová et al. 2022; Nakata et al. 2003; Vasseghian et al. 2021).

Because of their low water solubility, PAHs and PCBs are present in water basins at trace and ultra-trace levels (Wolska et al. 1999), which makes their determination challenging. Robust analytical methods have thus been developed to accurately isolate, enrich, and detect them in environmental aqueous matrices (Mwanza et al. 2021; Wolska et al. 1999; Wu et al. 2021).

In fact, analytical techniques suitable for PAH and PCB quantification at very low concentrations are fundamental according to current legislation protocols (Pellicer-Castell et al. 2022; Vasseghian et al. 2021).

Solid-phase extraction (SPE) is commonly used to enrich PCBs and PAHs before analysis because it requires low amount of solvents and allows good sample enrichment (Simsek et al. 2021). Other similar methods have been applied for the same purpose, such as solid-phase microextraction (SPME) (Baktash and Bagheri, 2017, Domínguez et al. 2018; Omarova et al. 2022) and stir bar sorptive extraction (SBSE) (Tankiewicz et al. 2011; Xiao et al. 2016). In addition, several sorbent materials (Nouri et al. 2020) with increasing selectivity and performing properties have been used such as hypercrosslinked polymers (Li et al. 2021), gold nanoparticles (Gutiérrez-Serpa et al. 2017; Pellicer-Castell et al. 2022), graphene oxide composites (Peng et al. 2022; Sheng et al. 2021; Song et al. 2022), and aerogels (Sun et al. 2022).

In this work, we propose a new analytical method for the preconcentration and quantitation of PCBs and PAHs in water samples based on SPE and thermal desorption in a pyrolysis–gas chromatography-mass spectrometry (Py-GC–MS) system. The SPE devices used are Magic Chemisorbers® (Frontier Lab) which consist of a polydimethylsiloxane (PDMS) stationary phase chemically bonded to a deactivated titanium tube. With chemisorbers, sample preparation is rapid, low-cost, efficient, environmentally friendly, and relatively easy. Our method was first validated using reference materials and subsequently tested on a water sample from the Morto Nuovo River (Pisa, Tuscany). To the best of our knowledge, this is the first application of Chemisorbers for the analysis of PAHs and PCBs in water matrix samples.

## Materials and methods

### *Chemicals*

Native PAH Stock Solution L429-PAR (purity > 98%, Wellington Laboratories, Canada): naphthalene, 2-methylnaphthalene, acenaphthylene, acenaphthene, fluorene, phenanthrene, anthracene, fluoranthene, pyrene, benz[a]anthracene, chrysene, benzo[b]fluoranthene, benzo[k]fluoranthene, benzo[e]pyrene, benzo[a]pyrene, perylene, indeno[1,2,3-c,d]pyrene, dibenz[a,h]anthracene, benzo[g,h,i]perylene.

Polychlorinated biphenyls’ list (purity > 98%, Labor. Dr. Ehrenstorfer, Russia): 4,4′-dichlorobiphenyl (PCB 15), 2,4′,5-trichlorobiphenyl (PCB 31), 2,4,2′,4′,5′-pentachlorobiphenyl (PCB99), 2,3,3′,4′,6-pentachlorobiphenyl (PCB110), 2,3,3′,5′,6-pentachlorobiphenyl (PCB 113), 2,2′,3,3′,4,6′-hexachlorobiphenyl (PCB132), 2,3,3′,4,4′,6-hexachlorobiphenyl (PCB 158); (from *Cambridge Isotope Laboratories* 35 µg/mL in isooctane): 2,2′,3,4,6′-pentachlorobiphenyl (PCB89), 2,2′,3,4′,5′,6-hexachlorobiphenyl (PCB149), 2,2′,3,5,5′,6-hexachlorobiphenyl (PCB151), 2,3,3′,4,4′,5-hexachlorobiphenyl (PCB156).

As an internal standard for PHA extraction and analysis, the L429-IS deuterated standard mix (purity > 98%, Wellington Laboratories) was used, while for the PCBs the mass-labeled MBP-MXE mix (purity > 99%, Wellington Laboratories) was used. The complete lists of the chemical species used as internal standards are reported in the Supporting Information.

2,2,4-Trimethylpentane (isooctane), pesticide grade, *Fluka;* Water LC–MS grade (Merck, Germany*).*

### Standard solutions

Standard and internal standard solutions were obtained by diluting the certified ones with LC–MS grade water. All the solutions were stored in a refrigerator at 4 °C. All reagents and chemicals were used without any further purification.

### Environmental sample pretreatment

The river “*Morto Nuovo*” borders the municipalities of San Giuliano Terme and Pisa, and its mouth is located within a national park that is recognized by UNESCO as an environmental biosphere reserve. Its hydraulic role, on the other hand, is due to the confluence of the channel of the wastewater and the water from the run-off of the cultivated fields in the northern part of the Pisan plain. Numerous untreated civil drainage channels enter its course which negatively impact on the river, making it of interest for environmental monitoring. The river is largely artificial, fed by drainage channels; therefore, it does not have a real source. Its flow gradually increases as it crosses the plain between two major rivers: the Serchio and the Arno (Figure S.1 in the Supporting Information).

The water sample was collected by lowering a stainless-steel metal container into the center of the water course, to avoid contamination from the banks. The sample was then filtered and stored in 1-L stainless steel containers for the analysis of organic compounds, which were then placed in a refrigerator at 4 °C, without any further purification. A field blank using LC–MS grade water was prepared and analyzed following the same procedure used on the water sample from the river.

### Extraction procedure

**Fig. 1:**
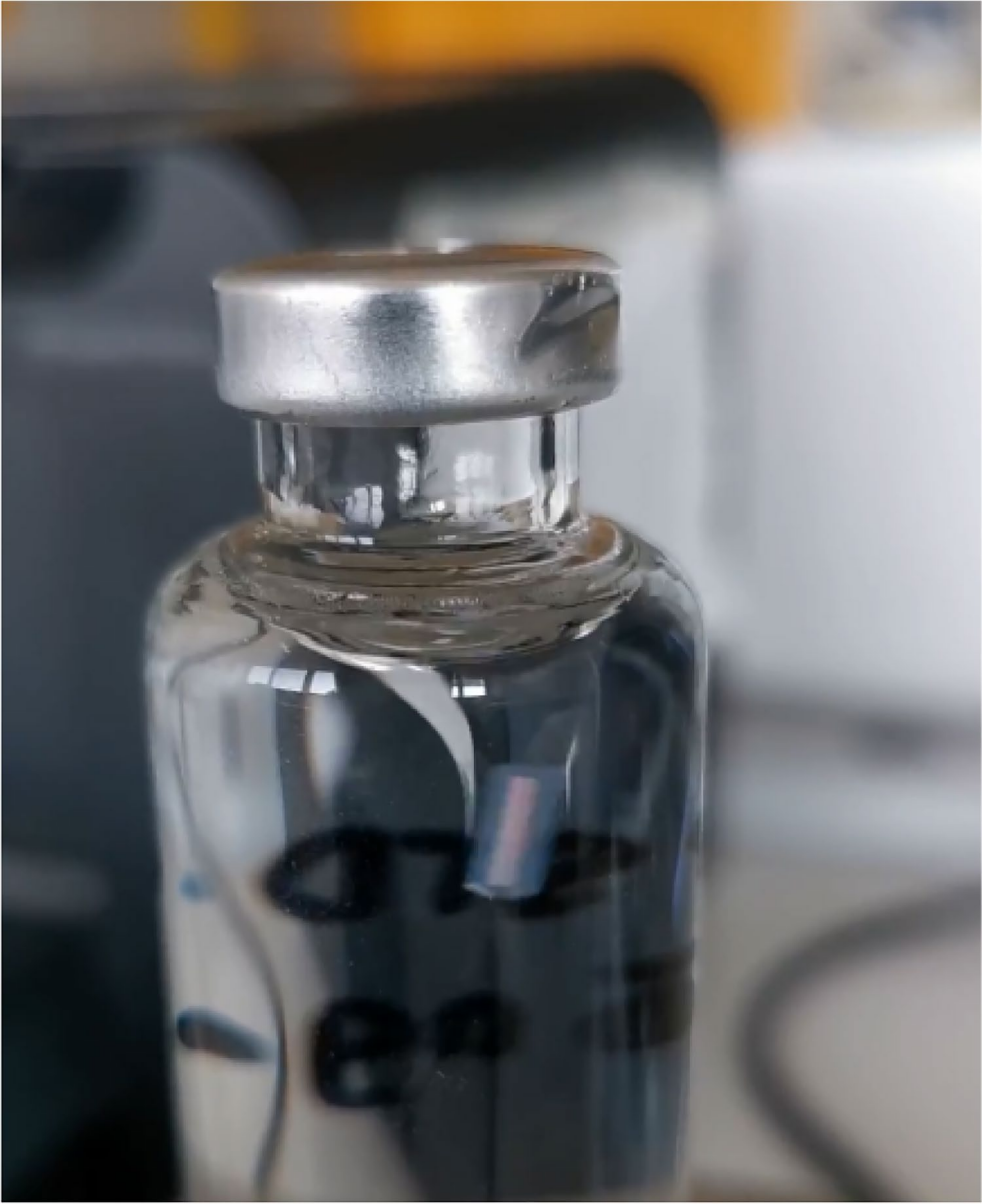
Chemisorber solid-phase extractor

The extraction of PAHs and PCBs was performed using a Magic Chemisorber® with a polydimethylsiloxane (PDMS) stationary phase. The Chemisorber is a solid-phase extraction device in which a film of 500 µm of PDMS is chemically bonded to the outer surface of a deactivated titanium tube (length 6 mm). For this preliminary study, all the solutions were subjected to extractions with the Chemisorber under stirring at 25 °C (Fig. 1) in a sealed vial at increasing extraction times (from 30 min to 8 h). Optimal conditions were obtained after 1 h of extraction time. The volume of the solutions/samples used for this study is 50 mL.

### Pyrolysis–gas chromatography-mass spectrometry equipment and conditions

The analyses were performed using a multi-shot pyrolyzer EGA/PY-3030D (Frontier Laboratories, Japan) coupled with an 8890 gas chromatograph, combined with a 5977B mass selective single quadrupole mass spectrometer detector (Agilent Technologies, US). The pyrolyzer system was equipped with a quick stabilizer pressure control QSP-1046E (Frontier Lab), and a Micro jet Cryo-Trap MJT-1035E (Frontier Lab). The cryo-focusing time was automatically controlled by the instrument software.

The Chemisorbers were desorbed in the pyrolysis system at 280 °C. The desorbed compounds were cryo-trapped with N_2_ at − 195 °C. Several desorption times were tested, and the best results were obtained at 15 min of desorption time.

The chromatographic separation of the pyrolysis products was performed on a fused silica capillary column HP-5MS UI (5% diphenyl–95% dimethyl-polysiloxane, 30 m − 0.25 mm i.d., 0.25-μm film thickness, J&W Scientific, Agilent Technologies), preceded by 2 m of deactivated fused silica pre-column with an internal diameter of 0.32 mm. The chromatographic conditions for the analysis were 35 °C held for 6 min, 20 °C/min to 310 °C held for 40 min. The helium (99.9995% purity) gas flow was set in constant flow mode at 1.0 mL/min. The mass spectrometer was operated in EI positive mode (70 eV, scanning m/z 35–700). Due to the use of a single quadrupole detector, we adopted a combined selected ion monitoring (SIM) and full scan method for the mass spectrometric acquisition. The SIM parameters are reported in Table S.1 in the Supporting Information.

## Results and discussion

### Method validation

**Fig. 2:**
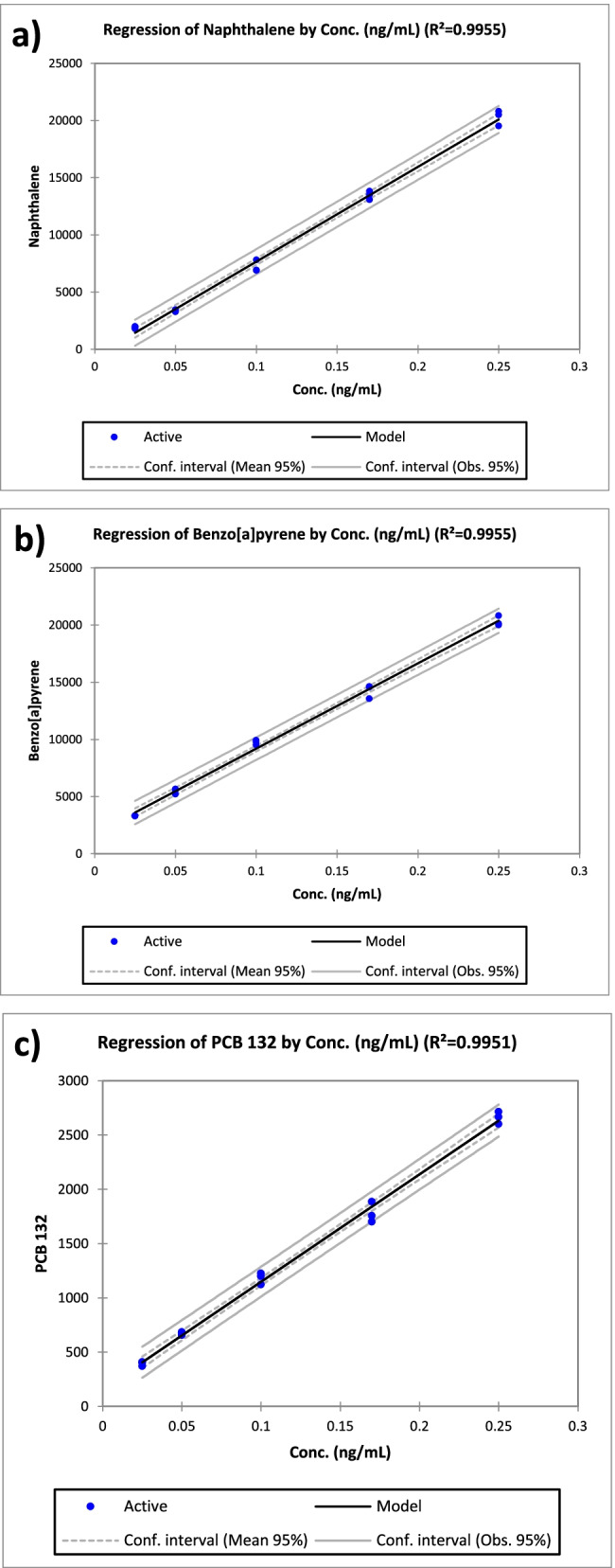
Calibration curves obtained for naphthalene, benzo[a]pyrene, and PCB132

The calibration curves for the 28 pollutants were obtained by diluting the concentrate standard solution in 50 ml of bidistilled water and adding the deuterated and mass-labeled internal standards mix for quality control and assurance. The curves were obtained in the range of 25–250 ng/L. Table 1 shows the equations obtained for PAHs and PCBs. Figure 2 shows the calibration curves obtained for a low and a high molecular weight PAH, naphthalene, and benzo[a]pyrene, and PCB132, respectively. All the curves were characterized by *R*^2^ values in the range of 0.9823–0.9975, showing a good linearity.

**Table 1:** Calibration curves, limit of detection (LOD), and quantitation (LOQ), intra- and interday coefficients of variation (CV%), and quantitative results obtained for the river sample

Analytes	Calibration curves			LOD *(*ng/L)	LOQ *(*ng/L)	Recovery (%)	Reproducibility		River sample (ng/L)
	Slope	Intercept	*R*^2^				CV% intra	CV% inter	
Naphthalene	82,887	− 628	0.9955	2.1	7.0	97	5.0	7.1	18
2-Methylnaphthalene	49,905	− 91	0.9969	1.9	6.3	100	4.2	7.2	17
Acenaphthylene	119,254	− 238	0.9970	2.3	7.7	97	4.5	6.5	< LOD
Acenaphthene	60,575	321	0.9907	2.5	8.5	101	5.0	7.2	9.0
Fluorene	134,092	304	0.9968	1.3	4.3	101	3.5	7.2	8.0
Phenanthrene	279,519	− 3044	0.9885	2.7	9.0	110	1.3	2.6	< LOD
Anthracene	291,934	− 3426	0.9883	1.8	6.0	108	1.7	0.9	< LOD
Fluoranthene	279,241	2414	0.9918	0.6	1.9	103	1.1	5.4	2.0
Pyrene	287,887	2592	0.9913	0.9	3.1	105	0.9	5.5	< LOD
Benz[a]anthracene	131,750	707	0.9965	2.4	8.1	102	1.7	2.8	< LOQ
Chrysene	285,155	− 20	0.9952	2.5	8.5	102	1.5	3.5	< LOD
Benzo[b]fluoranthene	32,777	550	0.9909	1.5	4.9	98	2.0	5.3	0.011
Benzo[k]fluoranthene	78,576	1883	0.9937	1.5	5.0	102	2.4	4.1	< LOQ
Benzo[e]pyrene	53,328	1932	0.9910	1.5	5.1	101	1.4	5.2	6.0
Benzo[a]pyrene	74,549	1741	0.9955	1.5	4.9	103	1.1	2.1	5.0
Perylene	100,716	1447	0.9950	1.5	5.1	98	0.6	4.3	7.0
Indeno[1,2,3-c,d]pyrene	409,025	− 1148	0.9892	1.1	3.7	104	1.6	2.2	< LOQ
Dibenz[a,h]anthracene	352,126	− 690	0.9872	1.6	5.4	96	4.1	6.0	< LOQ
Benzo[g,h,i]perylene	405,676	2878	0.9823	1.4	4.6	104	0.5	3.1	< LOQ
PCB 15	126,024	1259	0.9918	1.5	5.1	100	2.3	6.0	5.0
PCB 31	90,579	− 233	0.9970	1.5	5.0	101	0.7	4.8	< LOQ
PCB 99	39,955	182	0.9947	0.9	2.9	99	4.1	5.0	1.4
PCB 89 + 113 + 110	96,210	− 1113	0.9959	0.9	3.1	97	1.1	2.6	4.0
PCB 158	20,085	171	0.9943	1.9	6.5	99	2.7	4.9	< LOD
PCB 149	19,413	67	0.9851	0.9	2.9	97	1.8	2.8	3.0
PCB151	16,981	162	0.9875	1.0	3.2	97	2.7	3.7	3.0
PCB 156	48,344	− 412	0.9975	1.0	3.2	99	1.6	3.3	< LOD
PCB 132	9894	160	0.9951	0.9	3.1	99	2.8	4.5	< LOQ

Limits of detection (LODs) and quantification (LOQs) of PAHs and PCBs were evaluated on water blanks according to the ICH guidelines. Table 1 reports the limits of detection and quantification obtained for the method validation.

For both low and high molecular weight PAHs, the LODs were in the range of 0.9 and 2.7 ng/L, while the LOQs were lower than 9.0 ng/L. The method limits obtained for both the classes of PAHs did not show any significant differences, excluding any possible discrimination associated with their different molecular weights.

The limits obtained for PCBs were in a similar range to those obtained for the PAHs, with values lower than 1.9 ng/L for the LODs and 6.5 ng/L for the LOQs.

Intraday and interday repeatability was evaluated on a diluted standard water solution with a final concentration of 100 ng/L for the analytes. The coefficients of variations obtained for all the 28 pollutants are reported in Table 1. For the PAHs, the intraday CV% was lower than 5.0%, while the interday values were lower than 7.2%. The values obtained for the PCBs were relatively lower than those of PAHs, with values below 4.1% for the intraday and 6.0% for the interday.

The method recovery was evaluated on a triplicate of a water solution added with the reference standard in order to obtain a final concentration of PAHs and PCB of 100 ng/L. The recoveries for all the species are reported in Table 1. All the chemical species were characterized by recoveries in the range of 92–110%. A comparison of the two classes of compounds revealed that the PCBs were characterized by slightly lower recoveries, with values in the range of 97–101%.

The features of the method are consistent with those reported in the current literature for the most recent and relevant solid-phase extraction approaches used for the quantification of PAHs and PCBs in the environment (Table 2) (Baktash and Bagheri, 2017, Domínguez et al. 2018; Li et al. 2021; Peng et al. 2022; Roostaie et al. 2018; Sun et al. 2021; Tian et al. 2019, 2022; Xiao et al. 2016).

**Table 2:** Comparison of the Chemisorber approach features with the latest and more relevant approaches based on solid-phase extraction reported in literature

Extraction approach	Analytes	Extraction modes	Analytical methods	LOD	Ref
Magic Chemisorber® (PDMS, 500-µm thickness)	PAHs + PCBs	Immersion	Py-GC–MS	0.9–2.7 ng/L	-
SPME based on a chitosan-crosslinked graphene oxide aerogel stationary phase	PAHs + PCBs	Immersion	Desorption in the GC–MS injector	0.02–1.28 ng/L	Peng et al. (2022)
Magnetic solid-phase extraction based on Fe_3_O_4_@SiO_2_@CTS nano adsorbent	PCBs	Immersion	Desorption in the GC–MS injector	20 ng/L	Tian et al. (2022)
SPME with superhydrophobic silica aerogel stationary phase	PCBs	Immersion	Desorption in the GC–MS injector	100–1200 ng/L	Baktash and Bagheri (2017)
SPME with organic–inorganic hybrid silica aerogel stationary phase	PAHs	Immersion	Desorption in the GC-FID injector	1.0–30 ng/L	Tian et al. (2019)
SPME with poly(ionic liquid)-hybridized silica aerogel stationary phase	PAHs	Immersion	Desorption in the GC-FID injector	1.0–10 ng/L	Sun et al. (2021)
NTD with triethylchlorosilane-modified nanoporous silica aerogel stationary phase	PCBs	Headspace	Desorption in the GC–MS injector	0.3–1.0 ng/L	Roostaie et al. (2018)
PDMS/MOF-coated stir bar	PCBs	Immersion	Injection in the GC-FPD after solvent recovery	48–220 ng/L	Xiao et al. (2016)
SPME with hypercrosslinked polymer (HCP) stationary phase	PAHs	Headspace	Desorption in the GC–MS injector	2.5–25 ng/L	Li et al. (2021)
SPME with polyacrylate stationary phase	PAHs + PCB	Headspace	Desorption in the GC-HRMS injector	0.05–5.0 ng/L	Domínguez et al. (2018)

### Extraction test on environmental samples

The Chemisorber was used to investigate a river water sample in order to evaluate the feasibility of the method for the characterization and quantification of PAHs and PCBs in real environmental samples. The concentrations of the pollutants detected are reported in Table 1. A field blank was prepared and pretreated as the environmental sample to evaluate the possible unforeseen presence of other contaminants during the method validation.

Interestingly, besides the 28 target analytes, the chromatograms were also characterized by the presence of other chemical species. The analysis revealed the presence of traces of several chemical species which we did not validate with our method and which were not present in the blanks. The analysis identified plasticizers, with diethyl phthalate, butyl isohexyl phthalate, and bis(2-pentyl) phthalate being the most abundant. These species are common additives used in the production of plastic objects. Together with these species, the analysis also highlighted the presence of butylhydroxytoluene and 2,4-di-tert-butylphenol, two antioxidant compounds generally used as additives in synthetic polymers. Finally, along with these chemical species, we found traces of aliphatic hydrocarbons (up to C_18_), and of different sterols, the most abundant being cholestadiene, cholesterol, and β-sitosterol.

The results obtained on the river sample led to the quantification of the 28 selected organic pollutants. More importantly, they highlighted how these solid-phase extraction devices can be exploited to study a larger number of analytes and enable several emerging organic contaminants, such as phthalate plasticizers and antioxidants, to be detected and quantified, in the same chromatographic run.

## Conclusions

We have presented the first application of the Magic Chemisorber® with a PDMS stationary phase for the extraction and quantification of PAHs and PCBs in freshwater samples. The results showed the potential of using this extraction device directly on small volumes of water, thereby avoiding the use of solvents. The analysis of the river sample highlighted how this approach can also be used to analyze emerging organic pollutants, such as phthalate plasticizers and antioxidants.

Our results show that this approach can be used to characterize organic pollutants in a Py-GC–MS system, thus broadening the range of applications of this instrumentation beyond the traditional environmental analyses (e.g., microplastics).

Our preliminary application of this device demonstrated that the method works well, with good linearity and reproducibly, and LODs are lower than 2.7 ng/L. Our results were comparable with recent results with other methods that use solid phases developed and tailored specifically for the analysis of PAHs and PCBs.

Finally, a further optimization of the extraction conditions and the possibility to use other mass spectrometric acquisition systems, such as triple quadrupole mass spectrometer, and implementation of specific chromatographic columns will further improve the overall performance of this approach.

## Supplementary Information

Below is the link to the electronic supplementary material.

Supplementary file1 (PDF 139 KB)

## Data Availability

The data that support the findings of this study are available from the corresponding author.
