# Supplementary material for: Magic extraction: solid-phase extraction and analytical pyrolysis to study polycyclic aromatic hydrocarbon and polychlorinated biphenyls in freshwater
Source: Environ Sci Pollut Res Int. 2022 Aug 8;29(42):64252–8. doi: 10.1007/s11356-022-22435-9 (PMC9477944; doi:10.1007/s11356-022-22435-9)
Supplement: Supplementary file 1 — Supplementary file1 (PDF 139 KB) [file 11356_2022_22435_MOESM1_ESM.pdf]

Magic extraction: solid phase extraction and analytical pyrolysis to study polycyclic aromatic hydrocarbon and polychlorinated biphenyls in freshwater

Jacopo La Nasa[1,2], Greta Biale[1], Francesca Modugno[1], Alessio Ceccarini[1], Stefania Giannarelli[1]

[1] Department of Chemistry and Industrial Chemistry, University of Pisa, Pisa, Italy
[2] National Interuniversity Consortium of Materials Science and Technology (INSTM), Florence, Italy

Supporting information

Composition of the deuterated and mass labeled internal standards:

Standard solution **L429-IS** (Conc. 100 µg/mL, Purity > 98%, Wellington Laboratories): Naphthalene-d8, 2-Methylnaphthalene-d10, Acenaphthylene-d8, Phenanthrene-d10, Fluoranthene-d10, Benz[a]anthracene-d12, Chrysene-d12, Benzo[b]fluoranthene-d12, Benzo[k]fluoranthene-d12,Benzo[a]pyrene-d12, Perylene-d12, Indeno[1,2,3-c,d]pyrene-d12, Dibenz[a,h]anthracene-d14, Benzo[g,h,i]perylene-d12.

Standard solution **MXE mix** (Conc. 5 µg/mL, Purity > 98%, Wellington Laboratories): 2,4,4'-Trichloro[13C12]biphenyl, 2,2',5,5'-Tetrachloro[13C12]biphenyl, 2,2',4,5,5'-Pentachloro[13C12]biphenyl, 2,2',3,4,4',5'-Hexachloro[13C12]biphenyl, 2,2',4,4',5,5'-Hexachloro[13C12]biphenyl, 2,2',3,4,4',5,5'-Heptachloro[13C12]biphenyl, Decachloro[13C12]biphenyl.

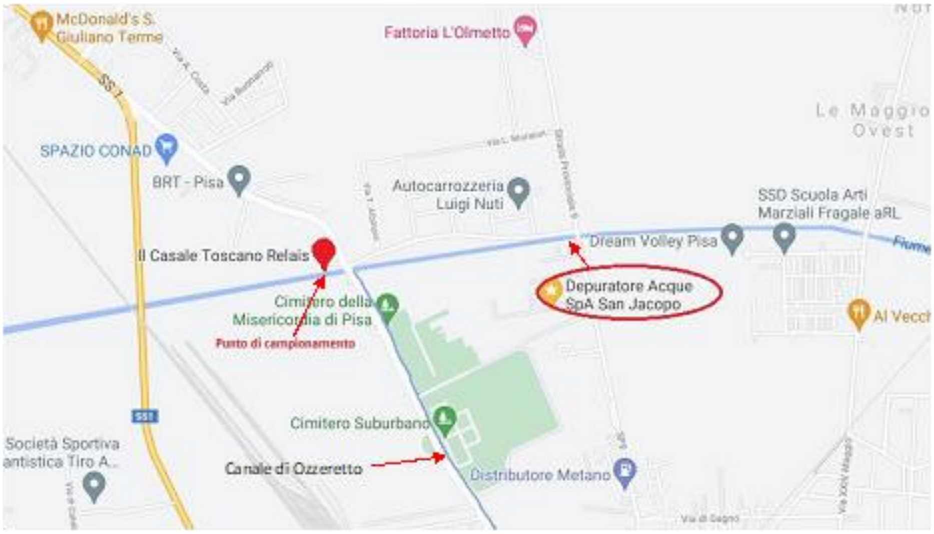

Figure S.1 – Sampling position of the environmental sample from Fiume Morto Nuovo (Pisa, Italy)

Table S.1 – Ions selected for the SIM GC-MS mass spectrometric acquisitions

Analytes	Time segment	Ions
naphthalene	0-8.50	128
2-Methylnaphthalene	8.5-13.3	142
acenaphthylene, acenaphthene	13.3-14.2	152
fluorene	14.2-15.3	166
phenanthrene, anthracene, PCB15	15.3-16.4	178, 222
PCB31	16.4-16.9	258
fluoranthene, pyrene, PCB89, PCB99, PCB110, PCB113	16.9-17.9	202, 326
PCB132, PCB149, PCB151, PCB156, PCB158	17.9-18.6	360
benz[a]anthracene, chrysene	18.6-19.5	228
benzo[b]fluoranthene, benzo[k]fluoranthene, benzo[e]pyrene, benzo[a]pyrene	19.5-21.1	252
indeno[1,2,3-c,d]pyrene, benzo[g,h,i]perylene, dibenz[a,h]anthracene	21.1-23.9	276,278
